# Levamisole-Contaminated Cocaine: An Emergent Cause of Vasculitis and Skin Necrosis

**DOI:** 10.1155/2014/434717

**Published:** 2014-03-20

**Authors:** Osama Souied, Hassan Baydoun, Zahraa Ghandour, Neville Mobarakai

**Affiliations:** ^1^Department of Internal Medicine, Staten Island University Hospital, 475 Seaview Avenue, Staten Island, NY 10305, USA; ^2^Faculty of Medical Sciences, Lebanese University, Beirut, Lebanon; ^3^Department of Infectious Diseases, Staten Island University Hospital, 475 Seaview Avenue, Staten Island, NY 10305, USA

## Abstract

The prevalence of cocaine adulterated with levamisole-induced vasculitis is increasing and physicians should be aware of this unique entity. There have been many reports of cutaneous vasculitis syndrome caused by cocaine which is contaminated with levamisole. Levamisole was used as an antihelminth drug and later was rescinded from use in humans due to adverse effects. Through this paper, we will report a 39-year-old crack cocaine user who presented with purpuric rash and skin necrosis of his ear lobes. Levamisole-induced vasculitis syndrome was suspected. A urine toxicology screen was positive for cocaine, opiates, and marijuana. Blood work revealed positive titres of ANA and p-ANCA, as well as anti-cardiolipin antibody. Biopsy taken from the left ear showed focal acute inflammation, chronic inflammation with thrombus formation, and extravasated blood cells. Treatment was primarily supportive with wound care.

## 1. Introduction

Levamisole, which was first developed as an antihelminth agent in the 1960s [1], can result in toxicity from the use of adulterated cocaine. It is an increasing reported cause of agranulocytosis, vasculopathy, and skin manifestations like specific rash and skin necrosis [2]. In this report, we describe the case of a 39-year-old crack cocaine user who presents with this unique thrombotic vasculitis, purpuric lesions, and skin necrosis of the ear lobes related to levamisole toxicity.

## 2. Case Presentation

A 39-year-old man with past medical history of cocaine abuse, gout, attention deficit hyperactivity disorder, and hand cellulitis secondary to methicillin-resistant* Staphylococcus aureus* (MRSA) infection presented with painful lesions on his right hand, left foot, and bilateral ears. Onset was three days prior to presentation where he started to have a constant burning sensation, most severely on the superior aspect of his ears. He had last smoked cracked cocaine one day prior to presentation and he was snorting it the day before.

**Figure 1:**
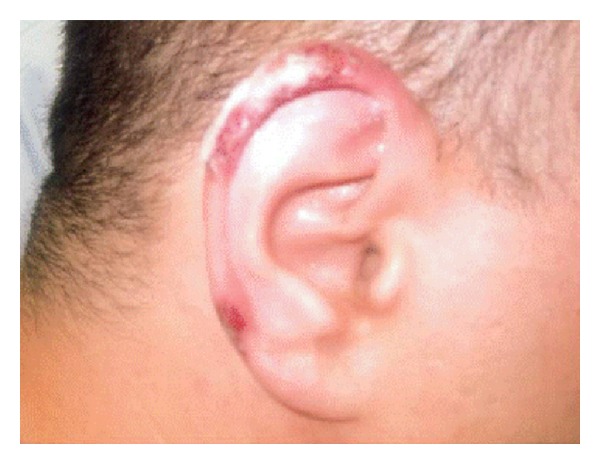
Tender and purpuric lesions with mild skin necrosis on the helical rim of the right ear.

On admission, the patient was afebrile, with blood pressure of 125/83 mmHg and heart rate of 110 beats per minute. On examination, the blisters on the dorsum of the right hand were new, although there was still an open wound from hand cellulitis secondary to MRSA infection 4 years ago on the dorsum of the second metacarpophalangeal joint. There was also a dry, closed, and scaly lesion on the left foot, as well as black necrotic bilateral auricular lesions with 1-2 mm blisters noted on both ears (Figure 1). The tongue had a hard nonerythematous nodule on the center, tender to touch. The rest of the physical examination was unremarkable.

Review of systems was negative for fever, chills, cough, hemoptysis, hematuria, Raynaud's phenomenon, alopecia, and oral or nasal ulcers. The patient had a history of necrotic lesions. They started 2 years ago, while at work he noticed dark patches on his cheeks and nose that would not wash off and were painful to touch. Over the next few hours, the patches spread bilaterally over the buccal area and the lower aspect of the nose. At that time he complained of fever, chills, myalgia, and joint pain. He was hospitalized and diagnosed with having septic vasculitis secondary to MRSA and was treated with vancomycin.

On this admission, laboratory testing showed a white count of 15.4 k/µL, hemoglobin of 14.9 g/dL, hematocrit of 41.9%, and platelet count of 208,000 k/µL. Basal metabolic profile, liver function tests, and haptoglobin were normal. Antinuclear antibody (ANA) and perinuclear-antineutrophil cytoplasmic antibody (p-ANCA) were weakly positive at a 1 : 40 titer and 1.1 U (normal <1 U), respectively. IGM cardiolipin antibody was positive at 19 U (normal <11 U). Antiphospholipid antibodies, complement level, HIV antibodies' titers, hepatitis panel, cytoplasmic antineutrophil cytoplasmic antibodies (c-ANCA), beta-2 glycoprotein, and blood and urine cultures were all negative. A urine toxicology screen was positive for cocaine, opiates, and marijuana. Biopsy taken from the superior aspect of the left ear showed focal acute inflammation of the surface epidermis, with foci of mild perivascular acute and chronic inflammation with thrombus formation and foci showing extravasated red blood cells. Burn and infectious disease services were consulted and recommended supportive wound care with bacitracin cream. The patient improved and was discharged few days later.

## 3. Discussion

Cocaine is the most commonly reported illicit drug in the emergency department in the United States [3]. There are around two million Americans who use cocaine on a regular basis. The Drug Enforcement Agency (DEA) reports that 69% of cocaine in the United States is contaminated with levamisole [4]. Later on and after the recognition of its immunomodulatory properties, levamisole was used in the treatment of different autoimmune diseases like ankylosing spondylitis and rheumatoid arthritis as well as various cancers [1, 5, 6]. It was withdrawn for use in humans in the United States in 1999 [2] due to its adverse side effects of agranulocytosis and vasculopathy [7].

Levamisole was first identified as a cocaine adulterant in the USA in 2003 and now it is found in around 70% of the cocaine seized in the United States as an adulterant [8]. The reason why levamisole is added intentionally during the manufacturing process of the cocaine is to potentiate the psychoaffective effects mainly by its stimulant activity due to dopamine release and also because of the similar appearance. It is usually detected by gas chromatography mass spectroscopy technique in urine specimens [9]. The first cases of levamisole-associated vasculitis and agranulocytosis among cocaine users were reported in 2008 in the southwestern United States [8]. Since that time, the number of cases continues to increase with more than 200 reported cases of levamisole related toxicity [4] which causes cutaneous, hematological, and neurological manifestations. The cutaneous involvement includes development of skin rash as purpuric papules, ecchymosis, and skin necrosis often having a “retiform,” “reticular,” or “stellate” pattern leading to ulceration and secondary infection and associated with leukopenia and autoantibody production [10]. Bilateral ear involvement, especially helical margins, is seen also in the majority of patients [11]. It was also described in some pediatric cases related to the use of levamisole in nephritic syndrome where the pathological exam revealed a mixed leukocytoclastic and thrombotic vasculitis, or a purely thrombotic vasculopathy [12].

Although cocaine contaminated with levamisole was previously reported to be associated with neutropenia or agranulocytosis [13, 14], our patient did not have these findings.

Another characteristic of the vasculitis related to levamisole is the association of autoantibodies production especially ANCA, anticardiolipin, and lupus anticoagulant antibodies [15]. Previous reports showed also that cocaine by itself can be associated with an ANCA positive vasculitis and pseudovasculitis with special specificity for human neutrophil elastase (HNE-ANCA) in cocaine-induced midline destructive lesions, as well as anticardiolipin antibody production [16–18]. It is not known for the moment if these antibodies, which are associated with cocaine and levamisole use, are pathogenic or bystanders produced by general immune reaction. After exposure to levamisole, these antibodies normalize within 2–14 months after discontinuation of the drug [10]. Our patient showed weakly positive titers of ANA and p-ANCA as well as positivity for anticardiolipin antibody.

The diagnosis of cocaine/levamisole-induced cutaneous vasculitis relies on the history, clinical findings and a positive urine toxicology test with a 2-3-day window as well as the detection of levamisole both serum and urine using gas chromatography and mass spectrometry with a short half life of 5.6 hours [19].

The approach to treat these patients differs between the reported cases. Steroids were used in some cases with unclear benefit. However, most of the cases were treated supportively with a self-limited course. Cocaine/levamisole should be withdrawn from all the patients. Extensive skin involvement and necrosis will need treatment in special burn unit as well as debridement, skin grafts, and reconstructive procedures.

## 4. Conclusion

The number of cases of cocaine contaminated with levamisole-induced vasculitis is increased rapidly. This diagnosis should be suspected in any patient who presents with purpuric rash or skin necrosis and associated neutropenia, agranulocytosis, or positive ANCA or anticardiolipin antibody.

## References

[1] Amery WKP, Bruynseels JPJM (1992). Levamisole, the story and the lessons. *International Journal of Immunopharmacology*.

[2] Chang A, Osterloh J, Thomas J (2010). Levamisole: a dangerous new cocaine adulterant. *Clinical Pharmacology and Therapeutics*.

[3] Merikangas KR, McClair VL (2012). Epidemiology of substance use disorders. *Human Genetics*.

[4] Metwally O, Hamidi M, Townsend L, Abualula H, Zaitoun A, Lall T (2013). The cocaine trail: levamisole-induced leukocytoclastic vasculitis in a cocaine user. *Substance Abuse*.

[5] Runge LA, Pinals RS, Lourie SH, Tomar RH (1977). Treatment of rheumatoid arthritis with levamisole. A controlled trial. *Arthritis and Rheumatism*.

[6] Christensen KD (1979). Treatment of seronegative spondylarthritis with levamisole: a double-blind placebo-controlled study. *International Journal of Immunopharmacology*.

[7] Czuchlewski DR, Brackney M, Ewers C (2010). Clinicopathologic features of agranulocytosis in the setting of levamisole-tainted cocaine. *American Journal of Clinical Pathology*.

[8] (2009). Agranulocytosis associated with cocaine use—four States, March 2008–November 2009. *Morbidity and Mortality Weekly Report*.

[9] Trehy ML, Brown DJ, Woodruff JT (2011). Determination of levamisole in urine by gas chromatography-mass spectrometry. *Journal of Analytical Toxicology*.

[10] Gross RL, Brucker J, Bahce-Altuntas A (2011). A novel cutaneous vasculitis syndrome induced by levamisole-contaminated cocaine. *Clinical Rheumatology*.

[11] Jacob RS, Silva CY, Powers JG (2012). Levamisole-induced vasculopathy: a report of 2 cases and a novel histopathologic finding. *American Journal of Dermatopathology*.

[12] Menni S, Pistritto G, Gianotti R, Ghio L, Edefonti A (1997). Ear lobe bilateral necrosis by levamisole-induced occlusive vasculitis in a pediatric patient. *Pediatric Dermatology*.

[13] Zhu NY, Legatt DF, Turner AR (2009). Agranulocytosis after consumption of cocaine adulterated with levamisole. *Annals of Internal Medicine*.

[14] Buchanan JA, Oyer RJ, Patel NR (2010). A confirmed case of agranulocytosis after use of cocaine contaminated with levamisole. *Journal of Medical Toxicology*.

[15] Rongioletti F, Ghio L, Ginevri F (1999). Purpura of the ears: a distinctive vasculopathy with circulating autoantibodies complicating long-term treatment with levamisole in children. *British Journal of Dermatology*.

[16] Neynaber S, Mistry-Burchardi N, Rust C (2008). PR3-ANCA-positive necrotizing multi-organ vasculitis following cocaine abuse. *Acta Dermato-Venereologica*.

[17] Fritsma GA, Leikin JB, Maturen AJ, Froelich CJ, Hryhorczuk DO (1991). Detection of anticardiolipin antibody in patients with cocaine abuse. *Journal of Emergency Medicine*.

[18] Wiesner O, Russell KA, Lee AS (2004). Antineutrophil cytoplasmic antibodies reacting with human neutrophil elastase as a diagnostic marker for cocaine-induced midline destructive lesions but not autoimmune vasculitis. *Arthritis and Rheumatism*.

[19] Kouassi E, Caille G, Lery L (1986). Novel assay and pharmacokinetics of levamisole and *p*-hydroxylevamisole in human plasma and urine. *Biopharmaceutics and Drug Disposition*.

